# Novel Thermophilic Species of *Limisphaera* Demonstrate Facultatively Anaerobic Mode of Life and Utilize a Wide Range of Carbohydrates

**DOI:** 10.3390/life16091543

**Published:** 2026-09-16

**Authors:** Olga A. Podosokorskaya, Tatyana G. Sokolova, Kseniya S. Zayulina, Alexandra A. Klyukina, Polina R. Klepikova, Andrei A. Novikov, Alexander Y. Merkel, Alexander G. Elcheninov

**Affiliations:** 1 Winogradsky Institute of Microbiology, Research Centre of Biotechnology RAS, 7/2 Prospekt 60-letiya Oktyabrya, 117312 Moscow, Russia; tatso2204@gmail.com (T.G.S.); zauylinakc@gmail.com (K.S.Z.); alexandra.a.popova@gmail.com (A.A.K.); klepikova.pol@gmail.com (P.R.K.); alexandrmerkel@gmail.com (A.Y.M.); elcheninov.ag@gmail.com (A.G.E.); 2 Department of Physical and Colloid Chemistry, Gubkin University, 65/1 Leninsky Prospect, 119991 Moscow, Russia; novikov.a@gubkin.ru

**Keywords:** *Verrucomicrobiota*, thermophiles, facultative anaerobe, Fe reduction, hydrolytic bacteria, GH51 glycosidase, Geyser Valley, Lake Baikal

## Abstract

Two novel facultatively anaerobic thermophilic members of the genus *Limisphaera* (*Verrucomicrobiota*), strains 4302-co^T^ and VF2^T^, were isolated from terrestrial hot springs of Geyser Valley (Kamchatka) and the Lake Baikal region, Russia. Cells were motile cocci, forming aggregates; strain 4302-co^T^ grew optimally at a range of 60–65 °C and pH 7.4 and strain VF2^T^ at 55 °C and pH 8.5. Both were chemoorganoheterotrophs utilizing mono-, di-, and polysaccharides, including cellulose, xylan, xanthan gum, arabinan, gluco-, and galactomannans. Both grew anaerobically by fermentation or by respiration with nitrite, nitrate, ferric citrate, or ferrihydrite; dissimilatory iron reduction is reported here for the first time within the phylum *Verrucomicrobiota*. Their genomes encoded more than 100 glycoside hydrolases and more than 15 polysaccharide lyases, indicating high hydrolytic potential. Additionally, the metabolic pathways of sugar catabolism, fermentation, and both aerobic and anaerobic respiration were reconstructed. A GH51 glycoside hydrolase of strain 4302-co^T^ phylogenetically related to endoglucanases was heterologously expressed and shown to be active at 70 °C with unusually broad specificity, hydrolyzing both β-1,4 and β-1,3 linkages. Based on phylogenomic analysis and phenotypic features, the strains are assigned to two novel species, for which the names *Limisphaera vallis* sp. nov. and *Limisphaera sibirica* sp. nov. are proposed.

## 1. Introduction

*Verrucomicrobiota* is a long-known, ubiquitous, and ecologically important bacterial phylum. It includes aerobes and anaerobes, as well as chemoorganotrophs, which utilize a wide range of polysaccharides and make up the vast majority of the phylum, and autotrophic methanotrophs, all representatives of which belong to the single family *Methylacidiphilaceae*. Discovered over 90 years ago, members of *Verrucomicrobiota* are still underrepresented in pure cultures. However, recently, this phylum has attracted renewed attention, in part driven by the potential benefits of *Verrucomicrobiota* members, namely, the ability to efficiently hydrolyze complex polysaccharides under extreme environmental conditions (low and high pH, elevated salinity, oligotrophic conditions, etc.) [1,2]. Thermophily provides additional advantages for biotechnological applications, including reduced risk of contamination, increased substances solubility, and higher process rates [3,4]. Nevertheless, only a few representatives of thermophilic verrucomicrobia are currently known. For example, methanotrophs of the genus *Methylacidiphilum* have been isolated from hot springs in Kamchatka, Italy, New Zealand, the Philippines, and the United States, forming a group of moderately thermophilic acidophiles. Among the chemoorganotrophic representatives, *Limisphaera ngatamarikiensis*, also isolated from a hot spring in New Zealand, is a moderately thermophilic alkalitolerant bacterium [5], and was later reclassified as the type genus of a new order called *Limisphaerales* [6]. We have also described another member of this order, *Fontisphaera persica*, the type species of the novel family *Fontisphaeraceae*, isolated from a terrestrial hot spring in the Lake Baikal region [6]. This bacterium was a moderately thermophilic neutrophile, utilizing preferentially mono-, di- and polysaccharides during aerobic growth or fermentation. As far as we know, no other thermophilic verrucomicrobia, besides *F. persica* and *Methylacidiphilum kamchatkense*, have been isolated from terrestrial hot springs in Russia. At the same time, other members of *Verrucomicrobiota*, and in particular of the order *Limisphaerales*, are found in the hot springs of the Lake Baikal region and Kamchatka [6,7].

The aim of this work was to isolate and investigate novel verrucomicrobia from geographically distant thermal springs in Russia. This study focused primarily on hydrolytic representatives capable of anaerobic respiration with different electron acceptors. As a result, two novel thermophilic representatives of the genus *Limisphaera*, *Limisphaera vallis* strain 4302-co^T^ and *L. sibirica* strain VF2^T^, were successfully obtained and described.

## 2. Materials and Methods

### 2.1. Sampling and Isolation

A mixed sample of water and pink-to-peach fouling was collected from a hot spring in Geyser Valley (N54°26′22″ E160°8′38″, pH 7.85, and 61 °C), Kamchatka, Russia, in 2023, in sterile 50 mL vials and transported to the laboratory. Inoculum (10%, *v*/*v*) was added to 15 mL Hungate tubes (Bellco Glass, Vineland, NJ, USA) filled with 5 mL aerobic modified Widdel medium [8] supplemented with xyloglucan (1 g L^−1^; Megazyme, Wicklow, Ireland) and the tubes were incubated at 65 °C. After 7 days of incubation, the growth of cells with various morphotypes, including numerically dominant small cocci, was observed. After several subsequent transfers at 65 °C on the same medium, followed by serial 10-fold dilutions, the small cocci were isolated in a pure culture as strain 4302-co.

A mixed sample of water, silty sediment, and plastic bag fragments suspended in the water was collected from a hot spring (N52°59′14.04″ E108°18′27.97″) issuing from a borehole at the Goryachinsk thermal water basin, Russia, in 2023, in a sterile 50 mL glass vial and transported to the laboratory. An aerobic enrichment culture was obtained from the sample (10% inoculum) with LDPE (10 g L^−1^) as the sole carbon and energy source. A medium of the following composition (g L^−1^) was used: NH_4_Cl (0.5); MgCl_2_·6H_2_O (0.165); CaCl_2_·2H_2_O (0.05); KCl (0.165); KH_2_PO_4_ (0.25); NaHCO_3_ (1.0); 1 mL of a trace element solution [9]; and 1 mL of a vitamin solution [10]. Stable enrichment was obtained after 7 transfers. To isolate a pure culture, 1 mL of the enrichment was inoculated to 10 mL of the same medium supplemented with starch (1 g L^−1^; Merck, Darmstadt, Germany). Strain VF2 was isolated from an enrichment culture by serial 10-fold dilutions. An initial enrichment culture was obtained on plastic, but in the present work, strain VF2 involvement in plastic degradation was not investigated.

The purity of the strains was verified by cultivation on an organic-rich medium containing glucose and yeast extract or peptone (2 g L^−1^) at various pH levels (6.0–9.0) and temperatures (37–65 °C) to reveal fast-growing organotrophs, as well as by analyzing the whole-genome sequence assemblies.

### 2.2. Phenotypic and Chemotaxonomic Characterization

Cell morphology and the effects of temperature, pH, and NaCl concentration on the growth of strains 4302-co^T^ and VF2^T^ were analyzed using the above-mentioned mineral media with starch or cellobiose as a substrate, respectively. The spectrum of tested substrates included xanthan gum (Kelco, Okmulgee, OK, USA), locust bean gum, karaya gum, carboxymethylcellulose (CMC), microcrystalline cellulose (Avicel), xyloglucan, dextran, starch, chitin, pectin, alginate, inulin, tragacanth (all from Sigma-Aldrich, St. Louis, MO, USA), dextrin (Merck, Darmstadt, Germany), galactan, galactomannan, glucomannan, mannan, arabinan, curdlan, lichenan, pullulan, levan, xylan, rhamnogalacturonan polygalacturonan, D-galacturonate, pachyman (all from Megazyme, Wicklow, Ireland), chondroitin sulfate, hyaluronic acid (both from Biosynth, Compton, UK), and agarose (Helicon, Moscow, Russia) added directly to the mineral medium before autoclaving (4 g L^−1^). Glucose, pyruvate, lactate, beef extract (all from Sigma-Aldrich, St. Louis, MO, USA), fructose, xylose, raffinose, arabinose, mannose, yeast extract, tryptone, peptone (all from Dia-M, Moscow, Russia), rhamnose (ReaChem, Moscow, Russia), maltose (Merck, Darmstadt, Germany), cellobiose, sucrose (both from Acros Organics, Geel, Belgium), lactose (AppliChem, Darmstadt, Germany), galactose (Serva, Heidelberg, Germany), methanol (ChimMed, Moscow, Russia), and ribose (Carl Roth, Karlsruhe, Germany) were added after autoclaving to a final concentration of 0.5 or 1 g L^−1^ from filtered stock solutions. Elemental sulfur (1 g L^−1^), sodium thiosulfate (10 mM), sodium sulfate (10 mM), sodium nitrite (2 mM), sodium nitrate (10 mM), sodium arsenate (5 mM), sodium selenate (5 mM), ferric citrate (10 mM), and ferrihydrite (50 mM) were tested as possible electron acceptors for anaerobic respiration. Sulfide formation was detected spectrophotometrically according to Trüper and Schlegel [11]. Nitrite and nitrate reduction were monitored by qualitative tests for nitrate/nitrite (Merck, Darmstadt, Germany). Arsenate reduction was assessed by the formation of the characteristic yellow precipitate of arsenic sulfide (orpiment). Fe(III) reduction was analyzed using the ferrozine method [12]. Autotrophic growth was tested in a H_2_/CO_2_ gas phase (4:1, *v*/*v*) under anaerobic conditions without electron acceptors and in the presence of elemental sulfur, sodium nitrite, or sodium nitrate.

Catalase and oxidase reactions were checked with 3% hydrogen peroxide (Sigma-Aldrich, St. Louis, MO, USA) and *N*,*N*,*N′*,*N′*-tetramethyl-*p*-phenylenediamine [13]. For the analysis of cellular fatty acids (CFAs) and quinones, the cells of strains 4302-co^T^ and VF2^T^ grown in liquid medium supplemented with starch (1 g L^−1^) were harvested at the late exponential growth phase. CFAs were determined as described earlier [14] but with a CP-Sil 88 column instead of an HP-5MS column. Isoprenoid quinones were determined as described earlier [15].

### 2.3. Genome Sequencing, Assembly, Annotation, and Phylogenetic Analysis

Genome sequencing and raw read processing were done as described earlier [16] with a single modification: NovaSeq X Plus (Illumina, San Diego, CA, USA) was used instead of NovaSeq 6000. Assembly of whole-genome sequences was performed as published elsewhere [17]. Completeness and contamination of the genomic assemblies were measured using CheckM v.1.2.2 with the “Bacteria” marker set [18]. The whole-genome indices (ANI, AAI, and dDDH) were calculated according to Larralde et al., Kim et al., Hölzer, and Meier-Kolthoff et al., respectively [19,20,21,22]. To construct a phylogenetic tree, the 120 bacterial single-copy conserved marker genes (bac120) were annotated, concatenated, and aligned using GTDB-Tk v2.7.1 [23]. The phylogenetic tree was built using IQ-TREE v.3.0.1 [24].

### 2.4. Genome Analysis

The genomes of the strains 4302-co^T^ and VF2^T^ were annotated with the NCBI Prokaryotic Genome Annotation Pipeline, PGAP, v.6.10 [25].

Genes encoding carbohydrate-active enzymes (CAZymes) were identified using dbCAN v.4 with all available tools [26]. A further BLAST v.2.6.0 search against the curated Swiss-Prot database [27] was performed for a more accurate prediction of putative enzyme activity.

Reconstruction of central carbon metabolism, sugar utilization, as well as energy conservation pathways was performed by searching the genes encoding proteins responsible for these processes as described earlier [28]. Proteins from different known iron reducers and *Planctopirus limnophila* were used as references for the detection of multiheme cytochromes [29] and components of bacterial microcompartments (BMCs) and BMC-associated enzymes [30], respectively.

Phylogenetic analysis of molybdopterin oxidoreductases was performed as follows: sequences belonging to the prokaryotic molybdopterin-containing oxidoreductase family with evidence at the protein level as well as seven putative dissimilatory molybdopterin-containing oxidoreductases encoded in the analyzed genomes of *Limisphaera* were aligned using Muscle v.5.2 [31]. The alignment was trimmed using trimAL v.1.4.1 [32] with a –gt option of 0.85. The tree was constructed in RAxML v.8.2.12 [33] with the PROTGAMMAILG model and 1000 rapid bootstrap replications as support values. Visualization of the phylogenetic tree was performed using iTOL v.7 [34].

### 2.5. Biochemical Characterization of the Glycosidase from the GH51 Family

#### 2.5.1. In Silico Analysis of GH51 Glycosidases

The domain architecture and the presence of signal peptide were predicted using InterProScan [35] and SignalP v.6. [36]. Phylogenetic analysis of glycoside hydrolases from the GH51 family was performed as described earlier [37].

#### 2.5.2. Cloning of the Lim81

The gene encoding protein MGA4643381.1 of strain 4302-co^T^ (named *lim81*, with a length of 2190 bp) was amplified using primers designed in this study (Table S1) and the DNA of *Limisphaera* sp. 4302-co^T^ as a template. PCR product was purified with the Cleanup Standard Kit (#BC022, Evrogen, Moscow, Russia). Cloning of *lim81* was performed using the aLICator LIC Cloning and Expression Kit (#K1251, Thermo Fischer Scientific, Vilnius, Lithuania) with vector pLATE51, which encodes the N-terminal 6xHis-tag and an enterokinase cleavage site according to the manufacturer’s instructions. The presence of cloned genes was verified by PCR with DNA from grown *E. coli* colonies as the template and using specific primers (LIC forward-sequencing primer 5′-TAATACGACTCACTATAGGG-3′ and LIC reverse-sequencing primer 5′-GAGCGGATAACAATTTCACACAGG-3′), and confirmed by subsequent Sanger sequencing (Evrogen, Moscow, Russia).

#### 2.5.3. Gene Expression and Enzyme Purification

For the expression of recombinant protein Lim81, the *E. coli* BL21 (DE3) strain carrying vector pLATE51::*lim81* was grown in 1.5 L LB medium supplemented with ampicillin, 100 μg mL^−1^, at 37 °C for 5 h to an optical density (λ = 600 nm) of 0.5. Gene expression was induced by the addition of 0.5 mM isopropyl β-D-1-thiogalactopyranoside (IPTG). After 16 h of incubation at 20 °C, cells were harvested by centrifugation (9000× *g*, 4 °C, 20 min) and resuspended in the following buffer: 50 mM TrisHCl (pH 7.8), 150 mM NaCl, 20 mM imidazole, and 0.1% (*v*/*v*) protease inhibitor PMSF (DiaM, Moscow, Russia). Cells were disrupted by sonication using SoniPrep (MSE, London, UK) at 4 °C and further centrifuged (13,400× *g*, 4 °C, 30 min). The supernatant, containing soluble protein Lim81, was purified using a fast protein liquid chromatograph (FPLC, Äkta Start, Cytiva, Marlborough, MA, USA). Metal affinity chromatography was performed on a 1 mL HisTrap HP column (Cytiva, Marlborough, MA, USA). The column was equilibrated with Ni-A buffer (50 mM Tris-HCl (pH 7.8), 0.5 M NaCl, 20 mM imidazole). The supernatant was adjusted to the column and it was washed with 10 column volumes (CV) of a Ni-A buffer. Protein elution from the column was performed by a linear gradient of imidazole ranging from 20 mM to 500 mM using Ni-B buffer (50 mM Tris-HCl pH 7.8, 0.5 M NaCl, 500 mM imidazole); gradient volume was 15 CV. Fractions containing Lim81 were desalted and concentrated using VivaSpin centrifugal concentrators with a 10 kDa MWCO (Sartorius, Göttingen, Germany) at 3000× *g*, at 4 °C, for 15 min.

The presence of the target proteins was confirmed by electrophoresis in a polyacrylamide gel [38]. Protein ladders #22610, #26614, and #26630 (Thermo Fischer Scientific, Vilnius, Lithuania) as well as G2058 (Servisbio, Wuhan, China) were used as protein standards. Protein concentrations were measured using the Qubit Protein Assay Kit (Thermo Fischer Scientific, Waltham, MA, USA) and Bradford method [39].

#### 2.5.4. Biochemical Characterization of Glycosidase Lim81

The endoglucanase activity of different Lim81-containing fractions was visualized on agarose (2% *w*/*v*) plates or zymogram gel containing 0.2% CMC as a substrate; hydrolysis zones were detected via Congo red staining [40,41].

Glycoside hydrolase activity was measured using the DNSA (dinitrosalicylic acid) assay [42]. The reaction mixture (600 μL) contained buffer (50 mM TrisHCl, 0.5 mM CaCl_2_, pH 5.0) and 0.3% substrate (β-glucan, lichenan, arabinan, arabinoxylan, xylan, arabinogalactan, galactan, mannan, pachyman, curdlan (all from Megazyme), CMC, Avicel, laminarin (all from Sigma-Aldrich, St. Louis, MO, USA), and agarose (Helicon, Moscow, Russia). The reaction mixture was preincubated at the respective temperature. Reactions were initiated by adding 100 μL of cell extract or purified enzyme solutions, followed by incubation in a Thermo Shaker TS-100C (Biosan, Riga, Latvia). The same reaction mixtures without protein were used as the controls. Aliquots of 300 μL of reaction mixture were taken at 0, 2.5, and 24 h. In each aliquot, the reaction was stopped by adding the 3,5-dinitrosalicylic acid reagent (1:1, *v*:*v*) and then the mixtures were incubated for 15 min at 98 °C, cooled down to room temperature, and the absorbance at 575 nm was measured with a SPECTROstar Nano spectrophotometer (BMG Labtech, Ortenberg, Germany). All measurements were made in triplicate.

Temperature dependence was measured at pH 5.0 with barley β-glucan as a substrate, using Thermo Shaker TS-100C at the temperature range of 22–95 °C. The influence of pH on activity of the glycosidase Lim81 was analyzed in the pH range of 2.5–10.5 using a 50 mM HEPES buffer (pH 2.5–5.0), 50 mM MES buffer (pH 5–6.5), 50 mM MOPS (pH 6.5–7.0), 50 mM HEPES buffer (pH 7.0–7.5), 50 mM TrisHCl (pH 7.5–9.1), and 50 mM CAPS (pH 9.1–10.5) at 70 °C.

The influence of metal ions, denaturing, and reducing agents was evaluated by incubation of the reaction mixtures with β-glucan as a substrate, containing a 1–5 mM range of each of the following agents: CaCl_2_, CuCl_2_, MgCl_2_, MnCl_2_, ZnCl_2_, Cr(SO_4_)_2_, dithiothreitol (DTT), Tween 80, urea, Triton-X100, and sodium dodecyl sulfate (SDS). The enzymatic activities were measured using the DNSA assay as described above.

One unit (U) of enzyme activity is defined as the amount of enzyme required to release 1 µg ml^−1^ of the reducing sugars (equivalent to glucose, mannose, galactose, or xylose) per minute. Specific activity was calculated as the enzyme activity per milligram of total protein (U mg^−1^ protein).

## 3. Results and Discussion

### 3.1. Phenotypic Properties

**Figure 1:**
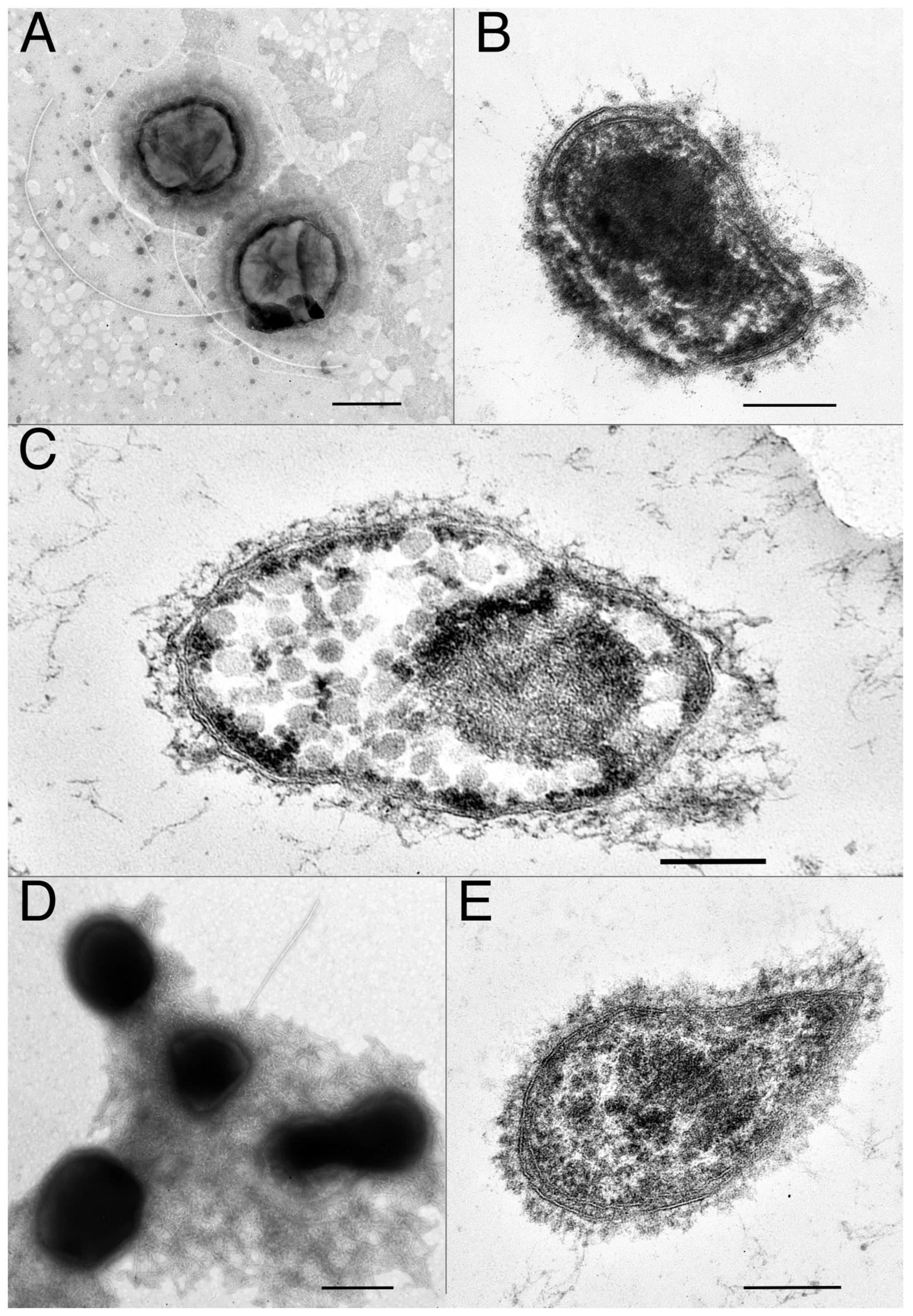
Transmission electron micrographs for the visualization of morphology and ultrastructures of novel isolates: (**A**) negatively stained cells of 4302-co^T^ with flagella, bar 0.5 µm; (**B**) thin section of the 4302-co^T^ cell grown on cellobiose, showing diderm (Gram-stain-negative) cell wall, bar 0.2 µm; (**C**) thin section of the 4302-co^T^ cell grown on rhamnose, showing bacterial microcompartments (BMCs), bar 0.2 µm; (**D**) negatively stained cells of VF2^T^ with flagellum in fibrillar matrix, bar 0.5 μm; (**E**) thin section of a VF2^T^ cell grown on sucrose, showing diderm (Gram-stain-negative) cell wall, bar 0.2 μm.

Cells of strains 4302-co^T^ and VF2^T^ were cocci with diameter ranges of 0.7–1.3 μm and 0.7–1.0 μm, respectively. Cells reproduced by binary fission. They were pink (4302-co^T^) or peach–white (VF2^T^) in color, and appeared singly, in pairs, or in aggregates. Aggregated cells of strain VF2^T^ were surrounded by a fibrillar matrix. Cells were motile in the exponential growth phase by means of a single flagellum (Figure 1A,D). Ultrathin sections of the cells revealed a diderm cell envelope (Figure 1B,E); strain 4302-co^T^ also possessed bacterial microcompartments (BMCs) when grown on rhamnose (Figure 1C).

Both strains were facultative anaerobes. Under aerobic conditions without shaking, the growth rate was slightly higher than under aerobic conditions with shaking or strictly anaerobic conditions. Catalase reactions were negative, while oxidase reactions were positive.

Strain 4302-co^T^ grew at a 37–70 °C range, with an optimum at the range of 60–65 °C, at a pH range of 6.2–9.2, with an optimum at 7.4. The strain grew in the presence of NaCl up to 1.5%; however, optimal growth occurred without NaCl in the medium. No growth was observed at temperatures ≤ 30 °C and ≥75 °C, at pH 5.9 and below and 9.7 and above, and at NaCl concentrations ≥ 1.9%.

The novel isolate 4302-co^T^ did not require yeast extract for growth. The following carbohydrates and proteinaceous substrates were utilized under aerobic and anaerobic conditions: glucose, arabinose, mannose, maltose, cellobiose, galactose, rhamnose, lactose, sucrose, xylose, ribose, raffinose, arabinan, dextrin, dextran, starch, xylan, xyloglucan, galactan, microcrystalline cellulose (Avicel), CMC, lichenan, pachyman, polygalacturonan, rhamnogalacturonan, glucomannan, galactomannan, xanthan gum, locust bean gum, chondroitin sulfate, yeast extract, beef extract, peptone, and tryptone. Fructose, alginate, agarose, levan, inulin, pectin, mannan, D-galacturonate, curdlan, hyaluronic acid, tragacanth, karaya gum, and pyruvate were not utilized. Strain 4302-co^T^ did not grow lithoautotrophically under an H_2_/CO_2_ atmosphere either in the presence or in the absence of electron acceptors (elemental sulfur, nitrate, nitrite). However, it grew organoheterotrophically by anaerobic respiration on cellobiose with sulfur, arsenate, nitrate, nitrite, ferric citrate, or ferrihydrite. Thiosulfate, sulfate, and selenate were not used for growth as electron acceptors. The products of cellobiose fermentation were acetate, lactate, ethanol, H_2_, and CO_2_. Major cellular fatty acids (>10%) of strain 4302-co^T^ were C_16:0_, anteiso-C_15:0_, and iso-C_16:0_ (Table S2). The only isoprenoid quinone was identified as MK-7.

Strain VF2^T^ grew at a 37–70 °C range, with an optimum at 55 °C, at a pH range of 6.5–9.5, with an optimum at 8.5. The strain grew in the presence of NaCl up to 1.5%; optimal growth occurred without NaCl in the medium. No growth was observed at temperatures ≤ 30 °C and ≥75 °C, at pH 5.9 and below and 9.7 and above, and at ≥1.9% of NaCl.

The novel isolate VF2^T^ was able to utilize yeast extract, as well as peptone, tryptone, and beef extract. The following carbohydrates were utilized under aerobic conditions: glucose, maltose, lactose, galactose, mannose, xylose, rhamnose, raffinose, fructose, sucrose, cellobiose, arabinose, arabinan, dextran, dextrin, starch, xylan, xyloglucan, galactan, CMC, microcrystalline cellulose (Avicel), glucomannan, galactomannan, rhamnogalacturonan, xanthan gum, karaya gum, locust bean gum, alginate, pectin, chitin, lichenan, pullulan, curdlan, pachyman, and polygalacturonan. Agarose and pyruvate were not utilized. Strain VF2^T^ grew anaerobically on glucose, sucrose, maltose, fructose, raffinose, galactose, starch, xylan, xanthan gum, and karaya gum, and did not grow anaerobically on pyruvate, lactate, or methanol. Strain VF2^T^ did not grow lithoautotrophically under an H_2_/CO_2_ atmosphere either in the presence or in the absence of electron acceptors (elemental sulfur, nitrate, nitrite). However, it grew by anaerobic respiration on sucrose, maltose, or starch with elemental sulfur, thiosulfate, nitrate or nitrite, ferric citrate, or ferrihydrite, and did not respire on sulfate, arsenate, or selenate. The products of glucose fermentation were acetate, ethanol, H_2_, and CO_2_. Major cellular fatty acids (>10%) of strain VF2^T^ were iso-C_16:0_, anteiso-C_15:0_, and anteiso-C_17:0_ (Table S2). The only isoprenoid quinone was identified as MK-7 similar to strain 4302-co^T^.

Thus, strains 4302-co^T^ and VF2^T^ shared several features with the only described member of the genus *Limisphaera*—*L. ngatamarikiensis* strain NGM72.4^T^. They were motile bacteria with spherical cells occurring singly or in pairs and dividing by binary fission; they had a diderm cell envelope; they were moderate thermophiles that grew optimally in the absence of sodium chloride; they were chemoorganotrophs that grew aerobically on simple and complex sugars and did not grow by means of sulfate respiration; and the sole detected quinone was MK-7 (Table 1). On the other hand, a number of features made them different from the closest relative *L. ngatamarikiensis*. They formed cell aggregates; they were facultative anaerobes able to grow by fermentation and anaerobic respiration with different electron acceptors, including nitrate; and they utilized a large number of polysaccharides (cellulose, xanthan gum, starch etc.), as well as proteinaceous substrates (yeast extract, peptone, etc.), but did not grow on pyruvate. In addition, strains 4302-co^T^ and VF2^T^ also differed from each other in a number of features: pigment composition, pH parameters, and the ability of strain VF2^T^ to grow on pectin, karaya gum, and fructose, as well as by anaerobic respiration with thiosulfate, but not with arsenate. Finally, all three strains were characterized by a different spectrum of major fatty acids.

### 3.2. Phylogeny and Analysis of Distribution

**Figure 2:**
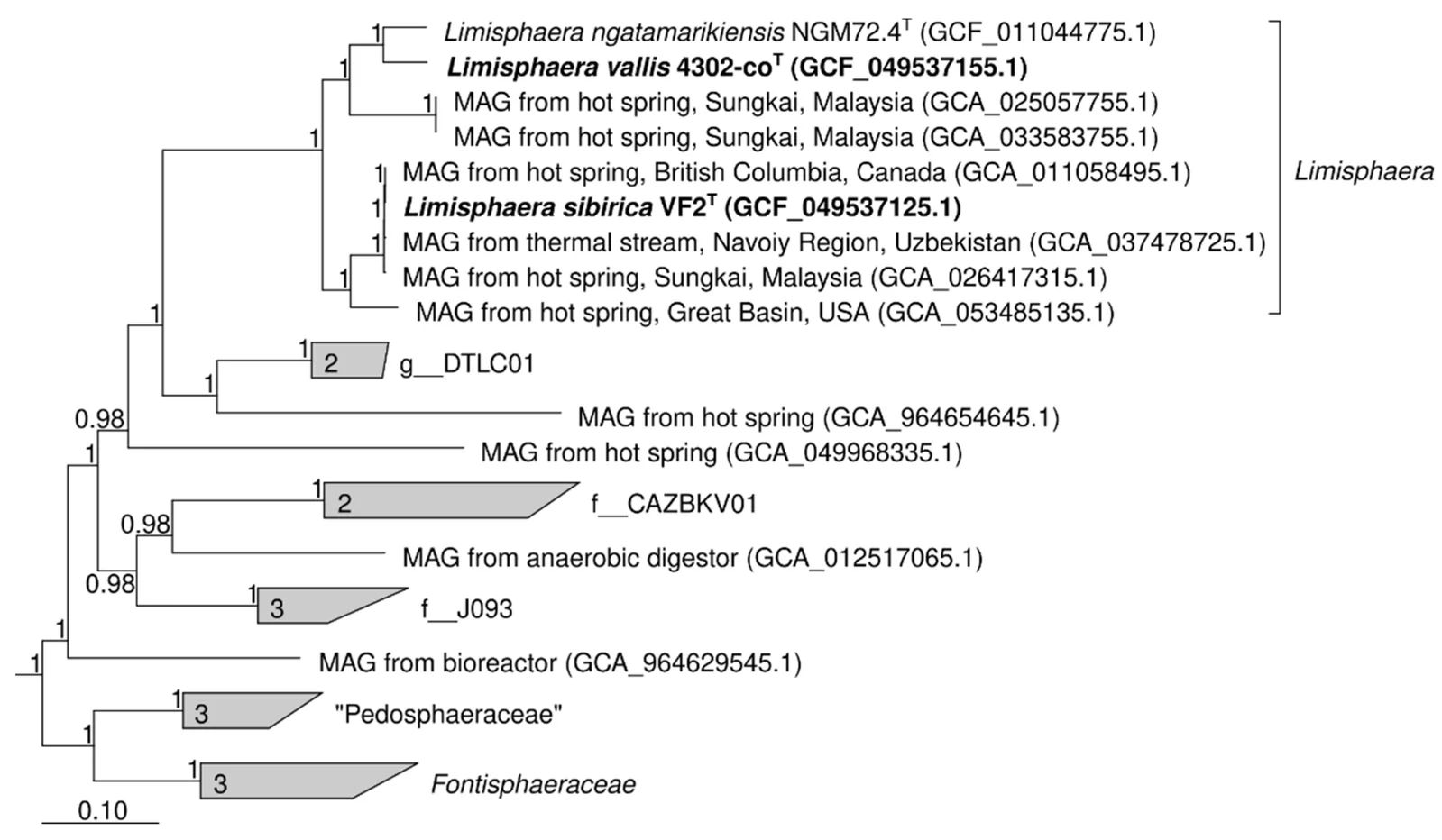
Phylogenetic placement of *Limisphaera vallis* 4302-co^T^ and *Limisphaera sibirica* VF2^T^ based on concatenated amino acid sequences of 120 bacterial single-copy conserved marker proteins. The length of the alignment is 20,811 aa. Bootstrap values are shown at the nodes. Bar, 0.1 change per position.

Both strains were first identified by 16S rRNA gene sequences extracted from the genomes. Strains 4302-co^T^ and VF2^T^ have 97.2% similarity to each other and 98.3% and 97.5% similarity to the closest cultured microorganism *Limisphaera ngatamarikiensis* NGM72.4^T^, respectively. ANI analysis also showed that genomes of 4302-co^T^ and VF2^T^ strains represent separate species within the genus *Limisphaera*: comparison of both strains with *L. ngatamarikiensis* yielded ANI values of 78.2 and 74.7%, respectively, and 74.7% between each other. Other results of the genomic comparison (dDDH and AAI) are given in Table S3. All these results clearly indicate that both strains represent separate species within the genus *Limisphaera*. Phylogenetic analysis based on 120 bacterial single-copy conserved marker genes [43] confirmed this conclusion (Figure 2). Therefore, based on phylogenomic analysis, we propose to assign strains 4302-co^T^ and VF2^T^ to novel species of the genus *Limisphaera*, *L. vallis* sp. nov., and *L. sibirica* sp. nov., respectively.

Species of the genus *Limisphaera* tend to thrive in terrestrial hot springs, and the species represented by the new strains are no exception. Using the Sandpiper service [44], we were able to reliably (coverage > 10×) detect species represented by strain VF2^T^ in 10 publicly available metagenomes. This species was detected in hot springs in Argentina (PRJEB67756), Canada (PRJNA375330), and Malaysia (PRJEB4990). In all cases, its relative abundance did not exceed 1.5%. The species represented by strain 4302-co^T^ was reliably detected in 14 publicly available metagenomes. Eleven of these metagenomes were obtained from hot spring sediments of Tengchong County, Yunnan Province, China [45], where its relative abundance was up to 1%. It was also detected in Tretyakovsky Hot Spring (Kunashir Island; PRJNA493162), but at a relative abundance of only 0.06%. In GenBank, we were able to find closely related MAGs only for strain VF2^T^. They share a 98.9–99.5% ANI range and were assembled from a hot spring in Sungkai, Malaysia [46], British Columbia, Canada (PRJNA480137), and a thermal stream in the Navoiy Region, Uzbekistan [47].

### 3.3. Genome Analysis

#### 3.3.1. General Genomic Features

The genomes of strains 4302-co^T^ and VF2^T^ were assembled into 37 and 14 contigs with N_50_ of 222,613 bp and 732,743 bp, respectively. The genome of strain 4302-co^T^ was 3.94 Mbp in size with a G + C proportion of 65.8%, while the length of the strain VF2^T^ genome was 4.32 Mbp with a G + C content of 65.2%. Both assemblies were of high quality with estimated completeness of 100% and contamination of 1.72%. According to the annotation, the genomes of strains 4302-co^T^ and VF2^T^ contained 2928 and 2957 protein-coding genes, respectively. A single rRNA operon was identified in each genome. Additionally, 46 tRNA genes, 4 ncRNA genes, and 29/24 pseudogenes were identified in the genomes.

In addition to the genomes of the two novel isolates, the genome of *L. ngatamarikiensis* and the available metagenome-assembled genomes (MAGs) affiliated with the genus *Limisphaera* were selected for further analysis.

#### 3.3.2. Diversity of Carbohydrate-Active Enzymes

**Figure 3:**
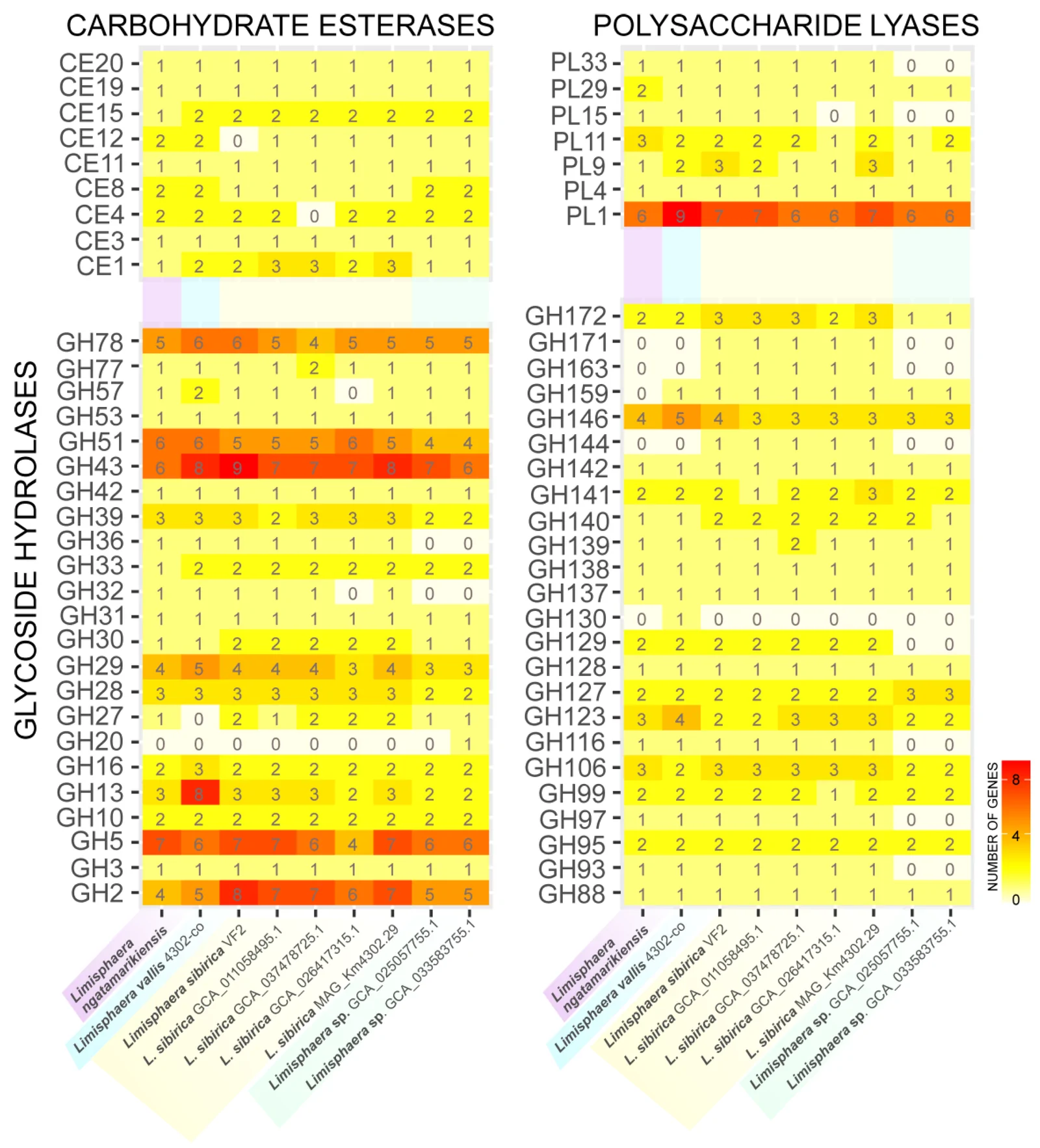
Distribution of CAZyme genes in the genomes of cultivated and uncultivated representatives of *Limisphaera* genus. Numbers in the heat map cells indicate gene counts from corresponding families of glycoside hydrolases (GHx), polysaccharide lyases (PLx), or carbohydrate esterases (CEx).

Since novel isolates, as well as all known members of the order *Limisphaerales*, are capable of utilizing a wide range of polysaccharides, the diversity of CAZymes in these bacteria and their functions were estimated. The genomes of the novel strains 4302-co^T^ and VF2^T^, as well as *L. ngatamarikiensis* and six MAGs, encoded a wide diversity of CAZymes; more than a hundred genes encoding glycoside hydrolases (GHs), polysaccharide lyases (PLs), and carbohydrate esterases (CEs) were found in each genome (Figure 3). Glycosidases from GH2 (5–8 genes), GH5 (4–7 genes), GH10 (2 genes), GH43 (6–9 genes), GH51 (4–6 genes), GH78 (4–6 genes), and GH146 (3–5 genes) and some other families were encoded in all genomes. These enzymes are presumably involved in the hydrolysis of plant polysaccharides, including xylans, arabinans, and galactans. Endo-acting enzymes are represented in GH5, GH10, GH51, GH53, and GH43 families and are able to hydrolyze cellulose, xylan, arabinan, galactan, gluco-, and galactomannans. The GH5 family exhibits a wide range of activities, including endoglucanase, xyloglucanase, endomannanase, and endoxylanase. The GH51 family encompasses endoglucanases, β-xylosidases, and α-L-arabinofuranosidases, the latter being involved in the hydrolysis of arabinans and arabinoxylans and often also possessing xylanase activity [48]. In the GH10 family, only endo-β-1,4-xylanases were found. The GH43 family includes α-L-arabinofuranosidases, α-L-arabinanases, β-D-xylosidases, and exo-α-1,3-galactanases. Glycosidases from the GH2 family were β-galactosidases and/or β-glucuronidases, while enzymes from the GH78 family presumably exhibited α-L-rhamnosidase activity. In addition, genes encoding polysaccharide lyases from families PL1, PL4, PL9, PL11, and PL29 were found in all genomes. Enzymes from these families exhibit pectin/pectate lyase (PL1, PL9) and rhamnogalacturonan lyase (PL4, PL9, and PL11) activities, which are essential for the degradation of pectins and their component, rhamnogalacturonan (RGN). Carbohydrate esterases, which are required for removing acetyl groups from xylan (CE1, CE3), pectin, and rhamnogalacturonan (CE20), as well as the methyl group in pectin (CE8, CE19), were also encoded in all genomes. The presence of a set of CAZymes involved in the degradation of hemicelluloses is consistent with the hydrolytic potential of *Limisphaerales* representatives and their ecological niche as degraders of plant-derived organic matter.

The genome of *L. sibirica* VF2^T^ encodes PL1, PL4, and PL9 (pectate and rhamnogalacturonan lyases), involved in pectin degradation, as well as PL15, a putative alginate lyase. The genome of *L. vallis* 4302-co^T^ encodes the majority of PLs, including PL1, PL4, and PL9 and PL11 (pectate and RGN lyases), PL29, and PL33 (both chondroitin/hyaluronate lyases), as well as pectin methyl esterases and RGN esterases belonging to CE8, CE19, and CE20 families, respectively. Moreover, both strains possessed β-xylosidases (GH3, GH5, and GH43), endo-1,4-β-xylanase (GH10, GH30), endoglucanases (GH5, GH16, GH51, GH128), endo-α-1,5-arabinases (GH43), and endo-β-1,4-galactanases (GH53). Genes encoding glycoside hydrolases from GH13 and GH57 families presumably active against α-glucans (starch, dextrin) were also present in the genomes of strains 4302-co^T^ and VF2^T^. The presence of numerous and variable CAZyme genes correlates with the wide spectrum of carbohydrate substrates utilized by these strains. This suggests the combined action of several enzymes towards recalcitrant polysaccharides (like cellulose, xanthan gum, and galactomannans) as well as involvement of specific sets of PLs, GHs, and CEs in the targeted degradation of acidic polysaccharides like pectin, RGN, and hyaluronate.

Comparison of CAZyme genes between the species (including MAGs) within the genus *Limisphaera* revealed a large common set among different species; however, some minor differences were revealed in their distribution (Table S4). Strain 4302-co^T^ lacked genes encoding α-galactosidases from the GH27 family; however, it had genes encoding the unique mannooligosaccharide phosphorylase (GH130) and eight genes of the GH13 family (compared with 2–3 genes in other genomes). The genome of strain VF2^T^ did not encode rhamnogalacturonan acetylesterase from CE12. On the other hand, strain VF2^T^ and MAGs belonging to the same species contained genes of endo-β-N-acetylglucosaminidases (GH163), which are involved in N-glycan hydrolysis [49,50], and exo-β-N-acetylmuramidases (GH171). MAGs SKYG106 and SKYGB_hs_bin94 had the narrowest repertoires of CAZyme genes with the absence of genes encoding β-fructosidases (GH32), some arabinan-active glycosidases (GH93, GH116), as well as polysaccharide lyases from PL15 and PL33 families. One β-hexosaminidase from the GH20 family was encoded only in MAG SKYGB_hs_bin94. This likely reflects a distinct ecological niche for this species, potentially driven by the limited availability of complex polysaccharides in its habitat.

#### 3.3.3. Sugar Transport and Central Carbon Metabolism

*Limisphaera* representatives can grow on various monosaccharides—products of polysaccharides hydrolysis. Transporters belonging to MFS and SSS families as well as ABC transporters from the CUT-2 family can be responsible for the import of mono-/oligosaccharides into cells. Surprisingly, all studied genomes contained several genes for the IIA subunit of the PTS; however, genes encoding other subunits were absent. These proteins may be involved in the regulation of carbohydrate metabolism rather than in transport processes [51].

**Figure 4:**
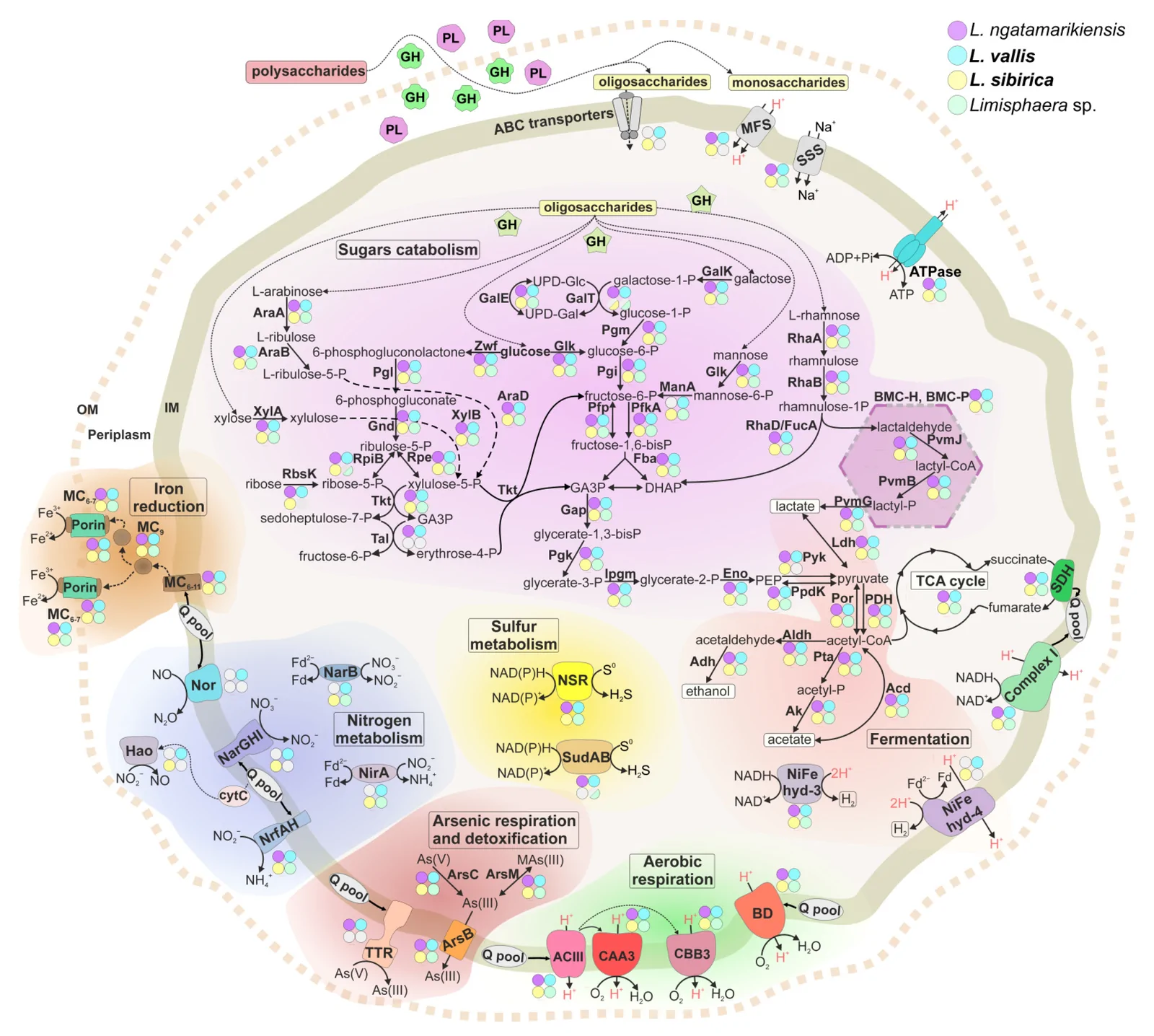
Schematic overview of metabolic processes reconstructed from *Limisphaera* genomes. Sugar catabolism: Glk—ROK family glucokinase, Pgi—glucose-6-P isomerase, PfkA/Pfp—ATP/pyrophosphate-dependent phosphofructokinase, Fba—class II fructose-bisphosphate aldolase, Gap—glyceraldehyde-3-phosphate dehydrogenase, Pgk—phosphoglycerate kinase, Ipgm—2,3-bisphosphoglycerate-independent phosphoglycerate mutase, Eno—enolase, Pyk—pyruvate kinase, PpdK—pyruvate, phosphate dikinase, Zwf—glucose-6-phosphate 1-dehydrogenase, Pgl—6-phosphogluconolactonase, Gnd—6-phosphogluconate dehydrogenase, RpiB—ribose-5-phosphate isomerase, Rpe—ribulose-phosphate 3-epimerase, Tkt—transketolase, Tal—transaldolase, GalK—galactokinase, GalT—galactose-1-phosphate uridylyltransferase, GalE—UDP-glucose 4-epimerase, Pgm—phosphoglucomutase, XylA—xylose isomerase, XylB—xylulose kinase, ManA—mannose-6-P isomerase, AraA—L-arabinose isomerase, AraB—ribulokinase, AraD—L-ribulose-5-phosphate 4-epimerase, RbsK—ribokinase, RhaA—L-rhamnose isomerase, RhaB—rhamnulokinase, RhaD/FucA—bifunctional rhamnulose-P/fuculose-P aldolase, PvmJ—lactaldehyde dehydrogenase, PvmB—lactyl-P transferase, PvmG—lactate kinase, Por—pyruvate:ferredoxin oxidoreductase complex, and PDH—pyruvate dehydrogenase complex. Fermentation: Ldh—lactate dehydrogenase, Pta—phosphate acetyltransferase, Ak—acetate kinase, Acd—ADP-forming acetate--CoA ligase, Aldh—acetaldehyde dehydrogenase, Adh—alcohol dehydrogenase, NiFe hyd—type 3 or type 4 NiFe hydrogenases. Aerobic respiration: ACIII—alternative complex III, BD—*bd*-type quinol oxidase, CBB3—*cbb3*-type cytochrome oxidase, and CAA3—*caa3*-type cytochrome oxidase. Nitrogen metabolism: NarGHI—dissimilatory nitrate reductase, NrfAH—dissimilatory nitrite reductase, Hao—hydroxylamine oxidoreductase-like enzyme, Nor—nitric oxide reductase, NarB—assimilatory nitrate reductase, and NirA—Fd-dependent assimilatory nitrite reductase. Iron reduction: MC_x_—multiheme cytochrome *c* with “x” heme-binding motifs. Arsenic respiration and detoxification: TTR—homolog of tetrathionate reductase with arsenate reductase activity, ArsC—cytoplasmic arsenate reductase, ArsM—arsenite methyltransferase, and ArsB—arsenite efflux permease. Sulfur metabolism: NSR—NAD(P)H sulfur oxidoreductase; SudAB—sulfide dehydrogenase.

Almost all studied genomes encoded the full set of enzymes of glycolysis for glucose utilization (Figure 4 and Table S5a) with the exception of two genomes belonging to uncultivated *Limisphaera* species, which lacked the pyruvate kinase genes; however, they had genes encoding pyruvate, phosphate dikinase indicating that glycolysis still may be active in these bacteria. Additionally, genes of enzymes involved in both oxidative (glucose-6-phosphate 1-dehydrogenase, 6-phosphogluconolactonase, and decarboxylating 6-phosphogluconate dehydrogenase) and non-oxidative branches of the pentose phosphate pathway were found. Ribose 5-phosphate isomerases in *Limisphaera* members had an additional serine hydroxymethyltransferase domain. BLAST search showed that such unusual enzymes are also present in different verrucomicrobia, including extremely acidophilic methanotroph “*Ca.* Methylacidiphilum infernorum” [52]. Surprisingly, strain VF2^T^ and the related MAGs, as well as species represented by only two MAGs (SKYG106 = GCA_025057755.1 and SKYGB_hs_bin94 = GCA_033583755.1), did not have transaldolase genes. These enzymes presumably can be functionally replaced with pyrophosphate-dependent phosphofructokinase, which produces sedoheptulose-1,7-bis-phosphate from sedoheptulose-7-phosphate, and fructose-1,6-bis-phosphate aldolase, which cleaves sedoheptulose-1,7-bis-phosphate to erythrose-4-phosphate and dihydroxyacetone phosphate, as has been proposed for some bacteria from *Bacillota* and *Planctomycetota* phyla [53,54]. All genomes except the *L. ngatamarikiensis* genome encoded mannose-6-phosphate isomerase required for mannose conversion into glucose. Ribokinase was encoded in all genomes with the exception of two MAGs, SKYG106 and SKYGB_hs_bin94. Genes encoding enzymes involved in the catabolism of L-arabinose (L-arabinose isomerase, ribulokinase, and L-ribulose-5-phosphate 4-epimerase) and galactose (galactose mutarotase, galactokinase, galactose-1-phosphate uridylyltransferase, and UDP-glucose 4-epimerase) were identified in all genomes. Probably, L-rhamnose is utilized through the action of rhamnose isomerase, rhamnulokinase, and rhamnulose-1-phosphate aldolase (Table S5b); further, several reactions associated with BMCs presumably occur as proposed for some representatives of *Bacillota* [55], *Planctomycetota* [30,56], *Verrucomicrobiota* [57], and *Bacteroidota* [58].

#### 3.3.4. Fermentation

Under anaerobic conditions without external electron acceptor *Limisphaera*, representatives can ferment organic substrates with the formation of several products (Table S5c). Phosphate acetyltransferase and acetate kinase are responsible for acetate production, while L-lactate dehydrogenase is responsible for lactate. Ethanol can be formed due to the presence of putative acetaldehyde dehydrogenase and both iron- and zinc-containing alcohol dehydrogenases. Probably, hydrogen is evolved through the action of [NiFe] hydrogenases of groups 3 and 4 but not [FeFe] hydrogenases, which are not encoded in the genomes.

#### 3.3.5. Aerobic and Anaerobic Respiration with Alternative Electron Acceptors

In the presence of electron acceptors in the medium, reducing equivalents are generated in the complete tricarboxylic acid cycle (Table S5a) and are further used in the respective electron transfer chains (ETCs). Both NADH:quinone oxidoreductase and succinate dehydrogenase transfer electrons to the quinone pool. Under aerobic conditions, several membrane complexes participate in respiration: *bd*-type quinol oxidase as well as *caa3*- and *cbb3*-type terminal cytochrome oxidases (Table S5d). No *Limisphaera* species possess the cytochrome *b*/*c1* complex, which is replaced by alternative complex III as described for *Rhodothermus marinus* [59].

Under anaerobic conditions, representatives of the genus *Limisphaera* can utilize different electron acceptors other than oxygen. All of them presumably can reduce nitrite due to the presence of genes encoding dissimilatory nitrite reductase NrfAH (Table S5e). At the same time, genes encoding distant homologs of membrane-bound nitrate reductase NarGHI (Figure S1) and putative hydroxylamine oxidoreductase were found only in genomes of strains 4302-co^T^ and VF2^T^ (as well as in MAGs affiliated to the same species). Nitric oxide reductase was a unique feature of strain 4302-co^T^.

Probably, homologs of NAD(P)H sulfur oxidoreductase are responsible for sulfur reduction in *Limisphaera* during “facilitated” fermentation as was previously proposed for *Thermotoga maritima* [60], while polysulfide or thiosulfate reductases were not encoded in any of the genomes (Table S5f). In addition, genomes of *L. ngatamarikiensis*, strain 4302-co^T^, as well as MAGs SKYG106 and SKYGB_hs_bin94 encoded sulfide dehydrogenase SudAB (MAG SKYGB_hs_bin94 had only *sudB*), which presumably performs NADPH-dependent sulfur reduction in cytoplasm [61].

Genes of the canonical arsenate oxidoreductase complex ArrABC were absent in all analyzed genomes; however, genes encoding another molybdopterin-containing oxidoreductase were found in the genome of *L. ngatamarikiensis* and strain 4302-co^T^ (Figure S1). Their closest characterized homologs were described as selenate reductase SdrABC [62] and tetrathionate reductase TtrABC [63]. Previously, it was proposed that some Ttr-related oxidoreductases may be responsible for the reduction of arsenate in bacteria and archaea [64,65,66]. Considering the growth experiments (the ability of strain 4302-co^T^ to grow with arsenate but not with selenate as an electron acceptor), we suggest that its Ttr-like complex (MGA4643419-21) acts as arsenate reductase. All analyzed *Limisphaera*-affiliated genomes also contained genes of proteins involved in arsenate detoxification (arsenate reductase ArsC, arsenite methyltransferase ArsM, and arsenite efflux permease ArsB).

Dissimilatory iron reduction presumably occurs due to the presence of several inner membrane, periplasmic, and porin-associated multiheme cytochromes *c* (MHCs) containing 6 to 11 heme-binding motifs (Table S5g) and encoded in several loci (1–3 genes). Two loci also contained genes of porins belonging to the OMR family not related to porins from well-studied Gram-stain-negative iron-reducer *Shewanella oneidensis* (MtrB). Moreover, one porin contains a single N-terminal heme-binding motif, as does MtrB in metal-reducing proteobacteria [67]. In another cluster, in addition to two inner membrane MHCs, three-subunit efflux transporters of the RND superfamily were encoded. The closest characterized homolog of the inner membrane subunit is the multidrug resistance protein MdtB, while the outer membrane subunit is relatively close to the OpmQ subunit of the exporter of pyoverdine, an iron siderophore specific for *Pseudomonas* [68]. Probably, members of *Limisphaera* could scavenge pyoverdine via this transporter, further export outside the cells and utilize it for iron chelation in the environment, or the identified transporter may serve an as yet unknown function.

Although some *Limisphaerales* representatives are considered methanotrophs based on genome analysis [69], methane monooxygenases and lanthanide-dependent enzymes, specific for known verrucomicrobial methanotrophs of the order *Methylacidiphilales* [70], were not encoded in the studied genomes of *Limisphaera*.

### 3.4. Biochemical Characterization of Glycosidase Lim81

The genomes of both novel isolates each contained five genes encoding glycoside hydrolases of the GH51 family. Most of these proteins in strains 4302-co^T^ and VF2^T^ are presumably alpha-L-arabinofuranosidases (Figure S2), which is a typical activity for GH51 enzymes. However, MGA4643381.1 (4302-co^T^) and MGA4578311.1 (VF2^T^) clustered with several characterized endoglucanases. Only 7 out of 121 glycoside hydrolases in this family are characterized as endoglucanases, and *Limisphaera* proteins are relatively distant from these homologs; therefore, MGA4643381.1 from *Limisphaera* sp. 4302-co^T^ was chosen for biochemical characterization. This enzyme possessed a Sec-type signal peptide (1–27), a family 8 carbohydrate-binding module (32–170), and GH51 family catalytic domain (219–599).

**Figure 5:**
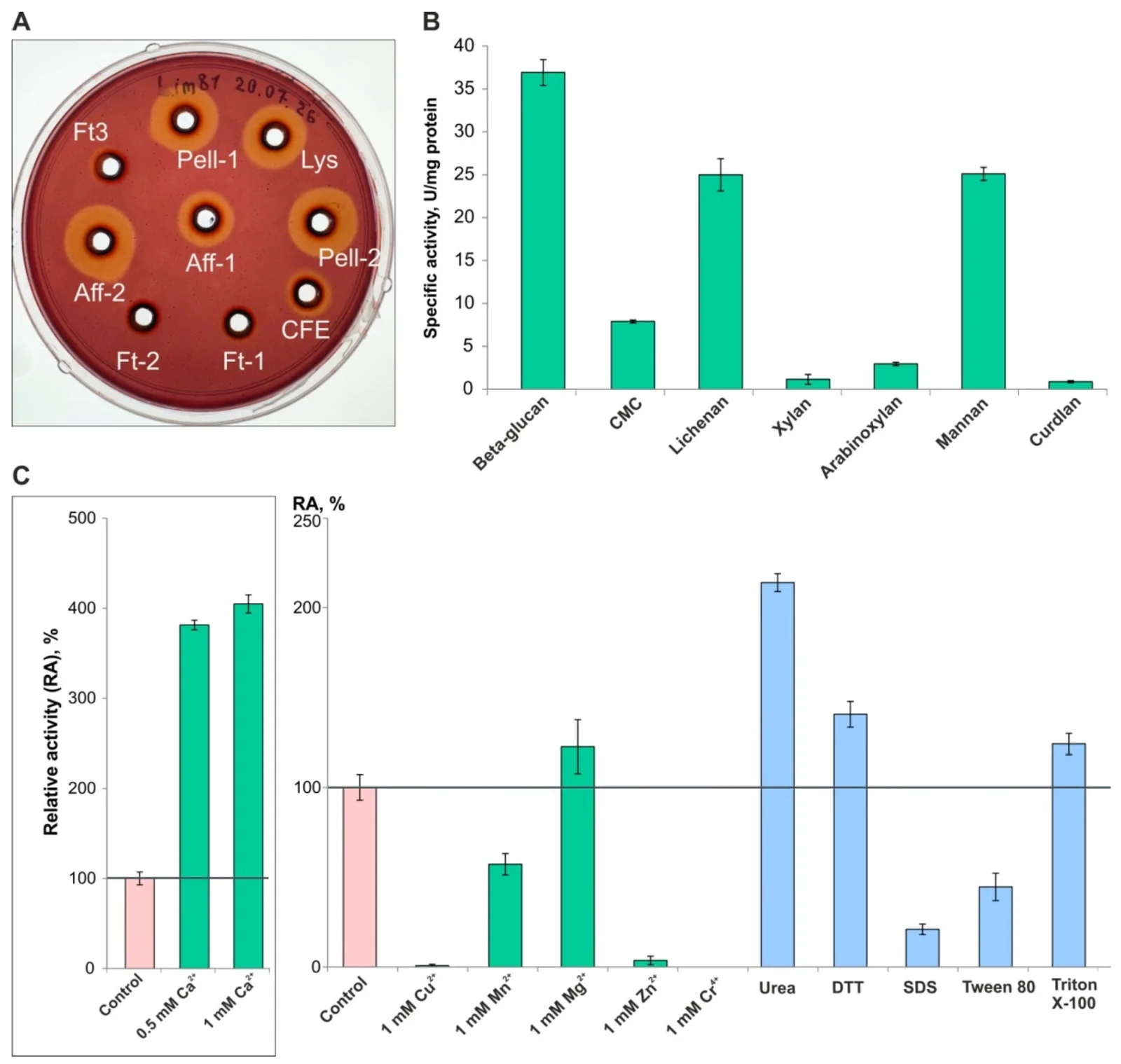
Enzymatic activities of GH51 glycosidase Lim81 from strain 4302-co^T^. (**A**) Endoglucanase activity towards CMC of fractions with Lim81; the plate was stained by 0.1% Congo red. Abbreviations: Lys—cell lysate after sonication, Pell-1, Pell-2—pellet with insoluble proteins, CFE—cell-free extract, Ft1—flow through fraction after application of CFE on HP column (unbinding proteins), Ft2—fraction of washed-from-affine-column unbound proteins, Aff-1, Aff-2—fractions with Lim81 protein (according Figure S3B), and Ft3—fraction of 500 mM imidazole, after column washing. (**B**) Substrate specificity of Lim81 towards different polysaccharides. Activity was measured at 70 °C and pH 5.0. Error bars represent the standard deviation of three replicates. (**C**) Influence of metal ions and reducing and denaturing agents on the activity of Lim81. Control—reaction mixture containing β-glucan without any additives (Me^+^ or detergents). Relative activity is presented as a percentage of the specific activity of the enzyme in the presence of ions/detergents.

The sequence length of the cloned part of *lim81* was 2190 bp (without signal peptide). The predicted mass of the protein was 80.5 kDa and pI 5.5. After heterologous expression and purification, a protein about 85 kDa was observed on SDS-PAGE (Figure S3A,B). Qualitative analysis of endoglucanase activity revealed the presence of active glycosidase in fractions of cell extract, pellet (containing insoluble proteins), and fractions from FPLC purification (Figure 5A and Figure S3C). Lim81 was active at pH 5.0 and 70 °C towards β-glucan, lichenan, mannan, and CMC with specific activities of 36.92, 25.0, 25.1, and 7.91 U mg^−1^ protein, respectively (Figure 5B). Weak activity was also observed against arabinoxylan, xylan, and curdlan (2.96, 1.16, and 0.88 U mg^−1^ protein). However, no activity was detected towards Avicel, amorphous cellulose, arabinan, arabinogalactan, galactan, pachyman, laminarin, and agarose. This substrate spectrum is consistent with the phylogenetic position of this enzyme within the clade of endoglucanases possessing broad specificity towards β-1,4-linked polysaccharides. Like the Lim81 enzyme, two endoglucanases of GH51, CelVA, and CelB families have also been shown to hydrolyze xylans [71,72]. The activity of Lim81 was stimulated fourfold by Ca^2+^ ions, while Mg^2+^ slightly increased it. Addition of Cu^2+^, Zn^2+^, and Mn^2+^ ions abolished or slightly reduced the activity (Figure 5C). Addition of urea, DTT, or Triton-X100 slightly increased the activity of Lim81, while the presence of Tween 80 and SDS inhibited the enzyme. This suggests that urea at low concentrations (1–5 mM) can act as an activator [73].

**Figure 6:**
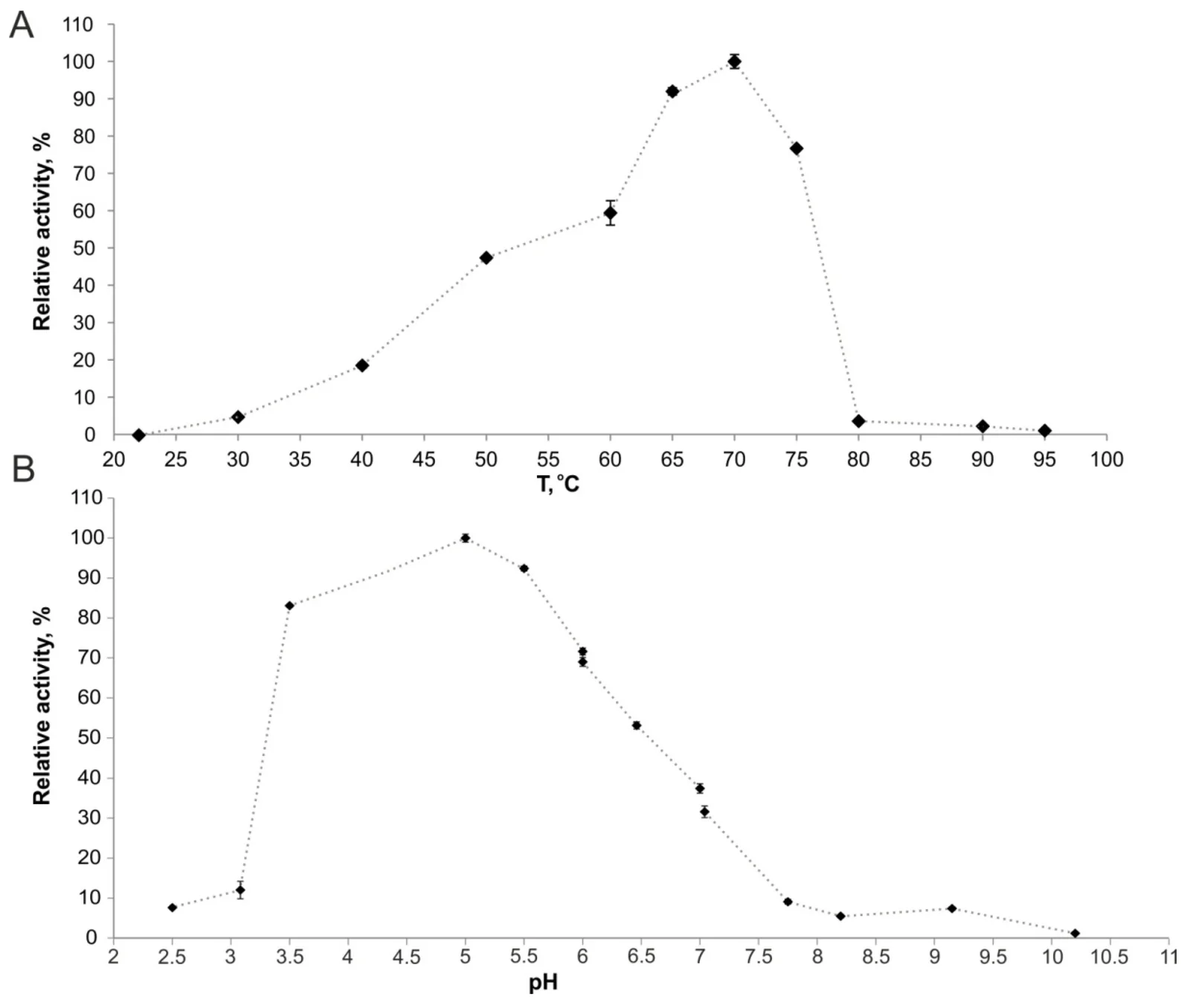
Effect of (**A**) temperature and (**B**) pH on the specific activity of recombinant Lim81. Activity was measured against β-glucan with the addition of 0.5 mM Ca^2+^.

Lim81 was active towards β-glucan at a temperature range from 30 to 80 °C (Figure 6A) with optimum at 70 °C, and a pH range of 3.5–8.5 with optimum 5.0 (Figure 6B). It is worth noting that only four endoglucanases from the family GH51 have been characterized to date: CelB, CelVA, CelA4, and Cel51A—three from the representatives of the genus *Alicyclobacillus* and the last one from *Fibrobacter* [71,72,73,74,75]. Also, the GH51-related protein, Fisuc_2081, isolated from *F. succinogenes* S85, possesses weak endoglucanase activity against β-glucan at 40 °C [75]. All these enzymes exhibit activity at low pH and thermophilic conditions (40–80 °C). Their primary activity is endoglucanase activity against CMC and β-glucan. For CelB, CelVA, and Cel51A, weak xylanolytic activity was also demonstrated (Table S6).

Among seven GH51-affiliated enzymes assigned as cellulases/endoglucanases, only five are biochemically characterized, while the function of SScel24 and Lc-CelC was predicted based on bioinformatic analysis (Table S6; [71,72,74,75,76,77,78]). Consequently, data on substrate specificity, stability, and characteristics of other GH51 cellulases are very scarce, and our data shed light on the ability and unique properties of these cellulases. Lim81 possessed an unusual ability to hydrolyze the β-1,4-linkages in various polysaccharides with a glucose or mannose backbone; weak activity against xylans and β-1,3-glucan (curdlan) was also observed. Future studies will focus on investigating the stability of this enzyme and its slight activation by detergents, while structural refinement and analysis should elucidate the mechanisms underlying the properties of this GH51 glycoside hydrolase.

## 4. Conclusions

In the current study, two strains, 4302-co^T^ and VF2^T^, were isolated from hot springs in Geyser Valley (Kamchatka) and the Baikal rift zone. Based on phylogenomic analysis, each strain was affiliated with the separate novel species of the genus *Limisphaera* (order *Limisphaerales* of the phylum *Verrucomicrobiota*). Both growth experiments and genome analysis revealed the strong hydrolytic potential of these bacteria as well as their metabolic flexibility, including aerobic and anaerobic respiration with different electron acceptors. The capacity for iron reduction was demonstrated here for the first time for the representatives of the phylum *Verrucomicrobiota*. Biochemical characterization of the glycosidase Lim81 from strain 4302-co^T^, which belongs to the family GH51, had broad specific activity against β-1,4-linkages in polysaccharides with a glucose, mannose, or xylose backbone, as well as weak β-1,3-glucanase activity. These bacteria were detected as minor components of microbiomes in diverse hot springs in Argentina, Canada, Malaysia, China, and Russia. Thus, thermophilic verrucomicrobia presumably play a crucial role in the processes of aerobic and anaerobic degradation of complex organic matter in terrestrial hot springs and can be promising objects for both the study of unknown physiological features and the discovery of new thermoactive enzymes, especially glycoside hydrolases.

### 4.1. Description of Limisphaera vallis sp. nov.

*Limisphaera vallis* (val’lis. L. gen. n. *vallis*, of a valley, here referring to Geyser Valley, Kamchatka, Russian Federation, from where the type strain was isolated).

Cells are cocci; 0.7–1.3 μm in diameter. Cells occur singly, in pairs, or in aggregates; some of them are motile by means of a single flagellum. They have a diderm cell envelope. Pink-pigmented under aerobic conditions. Moderate thermophiles with a growth temperature range of 37–70 °C and an optimum at 60–65 °C. The pH range for growth is 6.2–9.2 with the optimum at 7.4. Sodium chloride and yeast extract are not required for growth. Facultative anaerobes. Catalase negative and oxidase positive. Chemoorganotrophic. Can utilize glucose, mannose, maltose, cellobiose, arabinose, xylose, galactose, rhamnose, lactose, sucrose, ribose, raffinose, arabinan, dextrin, dextran, starch, xylan, xyloglucan, galactan, microcrystalline cellulose (Avicel), carboxymethylcellulose, lichenan, pachyman, polygalacturonan, rhamnogalacturonan, glucomannan, galactomannan, xanthan gum, locust bean gum, chondroitin sulfate, yeast extract, beef extract, peptone, and tryptone. No growth is observed on fructose, alginate, agarose, levan, inulin, pectin, mannan, monogalacturonate, curdlan, hyaluronic acid, karaya gum, tragacanth, pyruvate, or H_2_/CO_2_. Fermentation products of cellobiose are acetate, lactate, ethanol, hydrogen, and carbon dioxide. Can respire with elemental sulfur, arsenate, nitrite, nitrate, ferric citrate, ferrihydrite, and oxygen. Major fatty acids are C_16:0_, anteiso-C_15:0_, and iso-C_16:0_. MK-7 is the sole respiratory quinone. The type strain has a genome size of 3.94 Mb and a G+C content of 65.8%. The type strain, 4302-co^T^ = CGMCC 1.18274^T^ = BIM B-2082^T^ = VKM B-3882^T^ = UQM 41853^T^, was isolated from a terrestrial thermal spring in Geyser Valley, Kamchatka, Russia.

### 4.2. Description of Limisphaera sibirica sp. nov.

*Limisphaera sibirica* (si.bi’ri.ca. N. L. fem. adj. *sibirica*, originating from Siberia, a region in northern Asia, Russian Federation.

Cells are cocci; 0.7–1.0 μm in diameter. Cells occur singly, in pairs, or in aggregates; some of them are motile by means of a single flagellum. They have a diderm cell envelope. Slightly peach-pigmented in aerobic conditions. Moderate thermophiles with a growth temperature range of 37–70 °C and an optimum at 55 °C. The pH range for growth is 6.5–9.5 with the optimum at 8.5. Sodium chloride and yeast extract are not required for growth. Facultative anaerobe. Catalase negative and oxidase positive. Chemoorganotrophic. Can utilize glucose, maltose, lactose, galactose, mannose, xylose, rhamnose, raffinose, fructose, sucrose, cellobiose, arabinose, arabinan, dextran, dextrin, starch, xylan, xyloglucan, galactan, carboxymethylcellulose, Avicel, glucomannan, galactomannan, rhamnogalacturonan, xanthan gum, karaya gum, locust bean gum, pectin, chitin, lichenan, pullulan, curdlan, pachyman, polygalacturonan, yeast extract, beef extract, peptone, and tryptone. No growth is observed on agarose, pyruvate, or H_2_/CO_2_. Fermentation products of glucose are acetate, ethanol, hydrogen, and carbon dioxide. Can respire with elemental sulfur, thiosulfate, nitrate or nitrite, ferric citrate, or ferrihydrite, but not with sulfate, selenate, or arsenate. Major fatty acids are iso-C_16:0_, anteiso-C_15:0_, and anteiso-C_17:0_. MK-7 is the sole respiratory quinone. The strain has a genome size of 4.32 Mbp and G+C content of 65.2%. The type strain, VF2^T^ = VKM B-3901^T^ = UQM 41924^T^, was isolated from a terrestrial thermal spring in Goryachinsk, Baikal region, Russia.

### 4.3. Emended Description of Genus Limisphaera

The description is based on that provided by Anders et al. [5], with the following amendments. The genus contains strictly aerobic and facultatively anaerobic species. The latter oxidize organic substrates with nitrate, nitrite, elemental sulfur, ferric citrate, or ferrihydrite as electron acceptors or ferment mono-, di-, and polysaccharides or proteins. The major fatty acids are C_16:0_, ai-C_15:0_, i-C_16:0_, ai-C_17:0_, and C_18:0_. The type species is *Limisphaera ngatamarikiensis*.

## Figures and Tables

**Table 1:** Phenotypic characteristics differentiating strains 4302-co^T^, VF2^T^, and *Limisphaera ngatamarikiensis* strain NGM72.4^T^ [5].

Characteristic	4302-co^T^	VF2^T^	NGM72.4^T^
origin	Water/fouling, hot spring, Geyser Valley, Kamchatka, Russia	Water/sediment, hot spring, Goryachinsk thermal water basin, Russia	Water/clay, hot spring, New Zealand
cell size, μm	0.7–1.3	0.7–1.0	0.5–0.8
color	Pink	Peach–white	Pink
aggregate formation	+	+	−
min/opt/max temperature, °C	37/60–65/70	37/55/70	45/60–65/71
min/opt/max pH	6.2/7.4/9.2	6.5/8.5/9.5	5.6/8.1–8.4/8.9
min/opt/max NaCl, %	0/0/1.5	0/0/1.5	0/0/0.8
catalase/oxidase	−/+	−/+	+/+
oxygen tolerance	Facultative anaerobe	Facultative anaerobe	Aerobe and microaerobe
**Growth on substrates:**			
yeast extract	+	+	−
peptone	+	+	−
tryptone	+	+	ND
fructose	−	+	+
starch	+	+	−
cellulose	+	+	−
xanthan gum	+	+	−
pectin	−	+	−
pyruvate	−	−	+
**Reduction of:**			
nitrate	+	+	−
**Major fatty acids (>10%)**	C_16:0_, ai-C_15:0_, i-C_16:0_	i-C_16:0_, ai-C_15:0_, ai-C_17:0_	C_16:0_, ai-C_15:0_, i-C_16:0_, ai-C_17:0_, C_18:0_
**G + C%**	65.8	65.2	65.6
**Genome size, Mb**	3.94	4.32	3.91

All strains are Gram-stain-negative, motile cocci with monotrichous flagella, reproducing by binary fission, occurring single or in pairs and characterized by the ability to grow aerobically on glucose, arabinose, sucrose, mannose, xylose, maltose, galactose, cellobiose, dextrin, and locust bean gum; the inability to reduce sulfate; and presence of MK-7 as a sole respiratory quinone. ND, no data available; +, positive growth or reaction; −, negative growth or reaction.

## Data Availability

The whole-genome sequences were deposited into GenBank in BioProject PRJNA1222819: JBMVQZ000000000 (4302-co) and JBMVQY000000000 (VF2). The strains were deposited at the China General Microbiological Culture Collection Center, Belarusian Collection of Nonpathogenic Microorganisms, All-Russian Collection of Microorganisms, and Collection of Unique and Extremophilic Microorganisms under numbers 4302-co^T^ = CGMCC 1.18274^T^ = BIM B-2082^T^ = VKM B-3882^T^ = UQM 41853^T^ and VF2^T^ = VKM B-3901^T^ = UQM 41924 ^T^, respectively.

## Supplementary Materials

The following supporting information can be downloaded at https://www.mdpi.com/article/10.3390/life16091543/s1. Table S1: Primers designed for the *lim81* gene. Specific sequences are underlined; start- and stop codons are bolded. Table S2: Cellular fatty acids of strains 4302-co^T^ and VF2^T^. Table S3: Genome-to-genome comparisons of three species of *Limisphaera*. Table S4: Glycoside hydrolases, polysaccharide lyases, and carbohydrate esterases encoded in the genomes of *Limisphaera* representatives. Table S5: Enzymes involved in carbon metabolism and energy conservation within *Limisphaera* representatives. Table S6: Characterized cellulases from GH51 family. Figure S1: Phylogeny of molybdopterin-containing oxidoreductase subunits. Figure S2: Phylogeny of glycoside hydrolases from GH51 family. Figure S3: (A) SDS-PAGE of fractions, obtained after affinity chromatography, (B) affinity chromatography process, and (C) hydrolytic activities visualized on CMC–zymogram gel.
