# Supplementary material for: Novel Thermophilic Species of Limisphaera Demonstrate Facultatively Anaerobic Mode of Life and Utilize a Wide Range of Carbohydrates
Source: Life (Basel). 2026 Sep 16;16(9):1543. doi: 10.3390/life16091543 (PMC13608581; doi:10.3390/life16091543)
Supplement: Supplementary file 1 [file life-16-01543-s001.zip › Limisphaera_Life_Supplementary_fin_v2.pdf]

Supplementary Table S1. Primers designed for *lim81* gene. Specific sequences are underlined; start- and stop codons are bolded.

**Gene *lim81* (coordinates: 82-2193)**
Forward: 5\`- GGTGATGATGATGAC**AAG**<br>CAGGCGCCCCTGGCCATCTACA -3\`<br>Reverse: 5\`- GGAGATGGGAAGTCA**TTA**<br>GGGCCGGGCAAGAGCA -3\`

Supplementary Table S2. Cellular fatty acids of strains 4302-co^{T} and VF2^{T}

Fatty acid	ECL CP-Sil 88*	4302-co^{T}**	VF2^{T}
i-C14:0	13.523	1.1	0.5
C14:0	14.000	1.0	ND
i-C15:0	14.521	ND	0.3
ai-C15:0	14.711	23.5	28.5
C15:0	15.000	0.1	0.5
i-C16:0	15.525	15.1	34.9
C16:0	16.000	37.2	8.6
i-C17:0	16.529	ND	0.4
ai-C17:0	16.702	9.3	23.1
C17:0	17.000	0.9	0.9
i-C18:0	17.530	1.4	0.7
C18:0	18.000	9.8	1.3
ai-C19:0	18.718	0.1	ND
C18:2 ω6,9c	19.415	0.3	0.3
C18:3 ω3,6,9c	20.353	0.2	ND
3OH-C16:0	22.512	0.2	ND

*ECL, equivalent chain length for the Agilent CP-Sil 88 column (highly polar cyanopropyl phase)

**Table cells are conditionally formatted to guide the eye (from green=1.0 through yellow=5.0 to red=50.0). Fatty acids values are reported as total ion current peak area percentages.

Supplementary Table S3. Genome to genome comparisons of three species of *Limisphaera*

**16S rRNA gene identity (%)**	*Limisphaera ngatamarikiensis* NGM72.4	*Limisphaera vallis* 4302-co	*Limisphaera sibirica* VF2
Limisphaera ngatamarikiensis NGM72.4	100	98.3	97.5
Limisphaera vallis 4302-co	98.3	100	97.2
Limisphaera sibirica VF2	97.5	97.2	100
**ANI (%)**	*Limisphaera*	*Limisphaera*	*Limisphaera*

	ngatamarikiensis NGM72.4	*vallis* 4302-co	*sibirica* VF2
Limisphaera ngatamarikiensis NGM72.4	100	78.25	74.68
Limisphaera vallis 4302-co	78.25	100	74.86
Limisphaera sibirica VF2	74.68	74.86	100
**AAI (%)**	*Limisphaera ngatamarikiensis* NGM72.4	*Limisphaera vallis* 4302-co	*Limisphaera sibirica* VF2
Limisphaera ngatamarikiensis NGM72.4	100	82.94	77.32
Limisphaera vallis 4302-co	82.94	100	77.13
Limisphaera sibirica VF2	77.32	77.13	100
**dDDH (%) Formula: 2 (identities / HSP length)**	Limisphaera ngatamarikiensis NGM72.4	Limisphaera vallis 4302-co	Limisphaera sibirica VF2
Limisphaera ngatamarikiensis NGM72.4	100	21.8	20.4
Limisphaera vallis 4302-co	21.8	100	20.4
Limisphaera sibirica VF2	20.4	20.4	100

Supplementary Table S6. Characterized cellulases from GH51 family

**Protein name**	**Assession number**	**Organism/Source**	**T (min/opt/max)**	**pH (min/opt/max)**	**Substrate specificity**	**Additional comments**	**Reference**
Cel51A/EgF	AAC45377.1	*Fibrobacter succinogenes* S85	40	5.3	CMC, barley b-glucan, weak on AMC, xylan		[76]
Fisuc_2081	ACX75668.1	*Fibrobacter succinogenes* S86	40	5.8	b-glucan		[75]
CelB	ACV57112.1	*Alicyclobacillus acidocaldarius* DSM 446	40//80//100	3//4//5	CMC,AMC, xylan	*Ca, Mg ions stimulates, Zn inhibits*	[72]
CelA4	ADI82825.1	*Alicyclobacillus* sp. A4	35//60//75	2.2//3.4//6.4	beta-glucan		[74]
CelVA	CEH24710.1	*Alicyclobacillus vulcanalis* DSM16176	80	3.6//4.5	CMC, barley b-glucan,lichenan, xylan		[71]
SScel24	AJ622825.1	Uncultured bacterium cel gene for putative cellulase				*only prediction*	[77]
Lc-CelC	AHL27896.1	leaf–branch compost metagenome				*only prediction*	[78]
**Lim81**		Limispaera vallis	30//70//80	3//5//8.5	CMC, barley b-glucan,lichenan, mannan, arbinoxylan, xylan, curdlan	*Ca, Mg, urea, DTT, triton X-100 increased activity*	this study

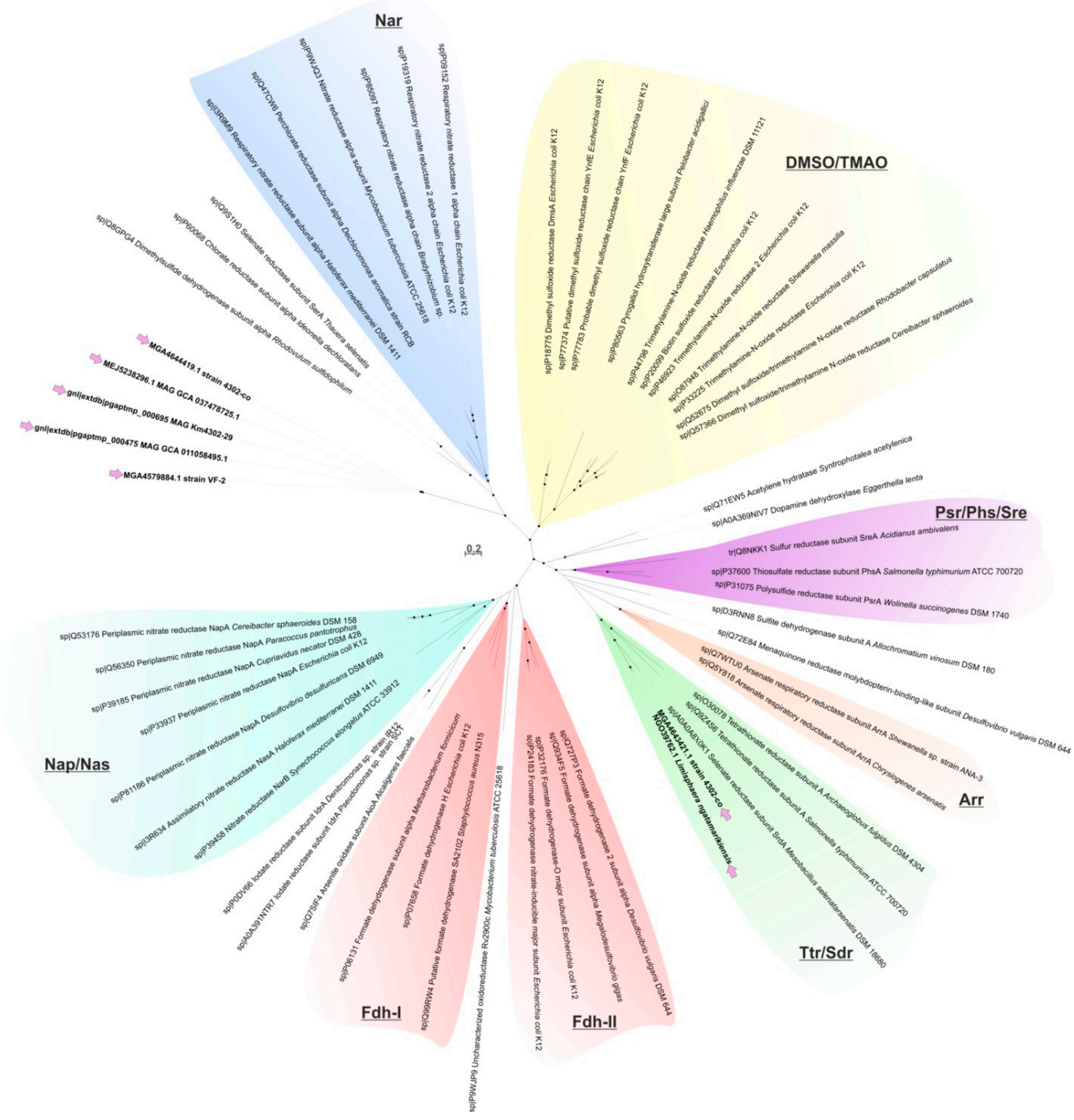

Supplementary Figure S1. Phylogeny of molybdopterin-containing oxidoreductase subunits: Psr/Phs/Sre – polysulfide reductase/thiosulfate reductases/sulfur reductase, Arr – arsenate reductases, Ttr/Sdr – tetrathionate reductases/selenate reducatse, Nar – respiratory nitrate reductases, DMSO/TMAO – dimethylsulfoxide/trimethylamine-N-oxide reductases, Fdh – formate dehydrogenases with I and II clades, Nap/Nas – periplasmic nitrate reductases/assimilatory nitrate reductases. Purple arrows marked proteins encoded in the genomes of *Limisphaera* representatives.

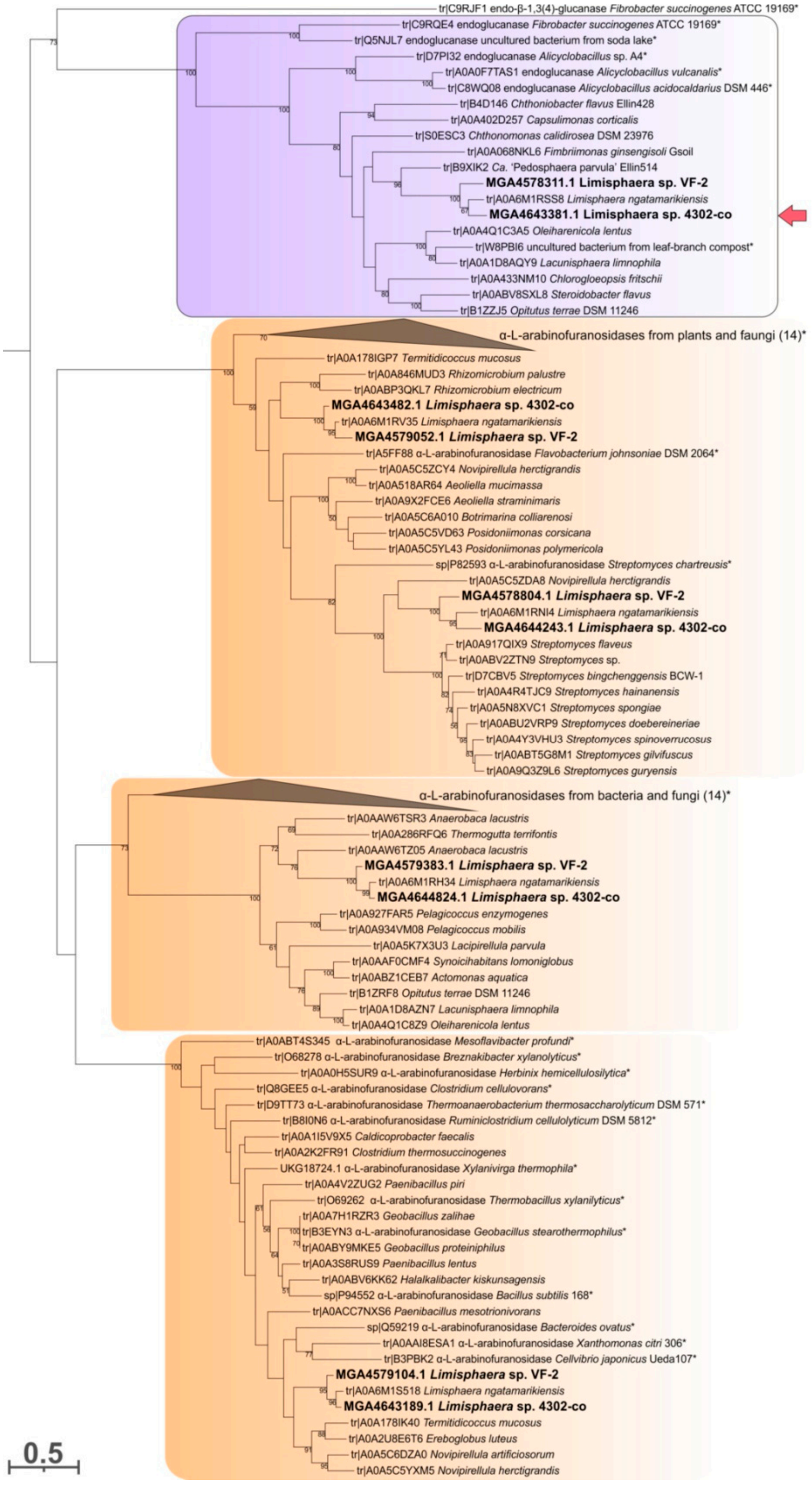

Supplementary Figure S2. Phylogeny of glycoside hydrolases from GH51 family (alpha-L-arabinofuranosidase and endoglucanase clades are into purple and orange boxes). Numbers at nodes indicate rapid bootstrap percentage values from 1000 repetitions (only values higher than 50% are shown). Biochemically characterized enzymes are marked with "*". Proteins encoded in the genomes of strain 4302-co and VF2 representatives are in bold; MGA4643381.1 selected for biochemical characterization is marked with red arrow.

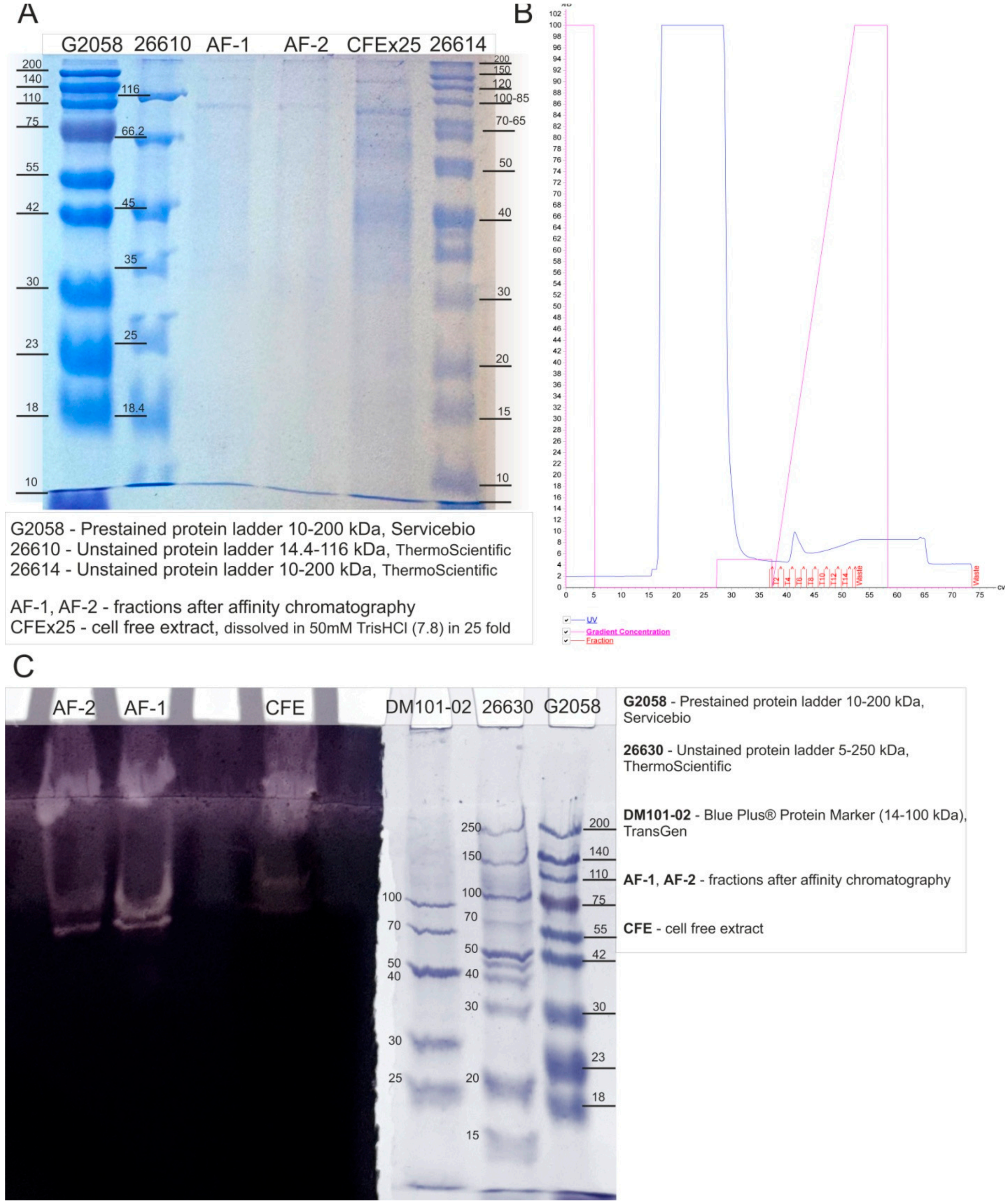

Supplementary Figure S3. (A) – SDS-PAAGE of fractions, obtained after affinity chromatography; **(B)** – Affinity chromatography (His Trap HP Ni-NTA agarose, 1 ml column, Cytiva); fractions 5,6,7 (total, 3 ml) were taken for further biochemical characterization; **(C)** – Hydrolytic activities visualized on CMC-zymogram gel. Following electrophoresis, incubation was performed in 50 mM MES 5.0 with CMC overnight (16 h) at 55°C and further staining of gel by Congo red.
